# Perspectives of Dermatology Program Directors on the Impact of Step 1 Pass/Fail

**DOI:** 10.7759/cureus.35801

**Published:** 2023-03-05

**Authors:** Peter Choi, Erik Langenau, Michael Roberts, Travis W Blalock

**Affiliations:** 1 Osteopathic Medicine, Philadelphia College of Osteopathic Medicine, Suwanee, USA; 2 Family Medicine, Philadelphia College of Osteopathic Medicine, Philadelphia, USA; 3 Statistics, Philadelphia College of Osteopathic Medicine, Philadelphia, USA; 4 Dermatology, Emory University School of Medicine, Atlanta, USA

**Keywords:** osteopathic, allopathic, residency, usmle, dermatology

## Abstract

Introduction: The shift of Step 1 to Pass/Fail has generated several questions and concerns about obtaining residency positions among allopathic and osteopathic students alike. Determining the perspectives of Dermatology Program Directors in regards to post-Step 1 Pass/Fail is critical for students to better prepare for matching into dermatology.

Methods: After receiving Institutional Review Board (IRB) exemption status, the program directors were chosen from 144 Accreditation Council for Graduate Medical Education (ACGME) and 27 American Osteopathic Association (AOA) Dermatology programs using contact information from their respective online website databases. An eight-item survey was constructed on a three-point Likert scale, one free text response, and four demographic questions. The anonymous survey was sent out over the course of three weeks with weekly individualized reminder requests for participation.

Results: A total of 54.54% of responders had “Letters of Recommendation” in their top 3. Forty-five percent of responders had “Completed Audition Rotation at Program” in their top 3. And, 38.09% of responders had “USMLE Step 2 CK Scores” in their top 3.

Conclusion: Approximately 50% of responders agreed that all medical students will have more difficulty matching dermatology. Based on the survey study, Dermatology program directors want to focus more on letters of recommendation, audition rotations, and Step 2 CK scores. Because each field seems to prioritize different aspects of an application, students should attempt to gain as much exposure to different fields such as through research and shadowing to narrow down their ideal specialties. Consequently, the student will have more time to tailor their applications to what residency admissions are looking for.

## Introduction

On their journey to becoming fully licensed physicians, nearly all medical students take the United States Medical Licensing Exam (USMLE) Step 1 Examination, which was implemented in the 1990s. Scores on the examination have traditionally been three-digit numerical values. In the spring of 2019, key organizations including the American Medical Association, the Association of American Medical Colleges, the Educational Commission for Foreign Medical Graduates, the National Board of Medical Examiners, and the Federation of State Medical Boards organized the Invitational Conference on USMLE Scoring to discuss shifting the scoring from the traditional three-digit numerical value to pass/fail [1-3].

This new policy was implemented in January 2022 and was introduced to alleviate the issues stemming from the three-digit scoring and to create positive changes in the medical educational system [3-7]. The numerical scoring was thought to place too much emphasis on rote knowledge while hindering the students’ ability to nurture other core competencies such as teamwork, empathy, and verbal skills. The shift in scoring also aimed to reduce the negative impact of a numerical value on underrepresented minority groups and increase representation and diversity in fields such as orthopedic surgery and dermatology that have traditionally been considered less diverse [3,8-10]. Furthermore, studies examining whether USMLE scores predicted success in residency have shown tenuous correlations, indicating that several other factors besides an examination score must be considered for predicting performance in residency [1,2,10].

In order to have some reprieve from the current issues mentioned above, residency program directors (PDs) will likely place greater importance on letters of recommendation, clerkship grades, research achievements, cultural competence, communication skills, and volunteer experiences, while simultaneously reducing the weight they give to standardized test outcomes. Several survey studies in various fields have asked PDs to indicate what they will be focusing on more since Step 1 has become pass/fail.

A survey study of otolaryngology PDs showed that around 68% of PD cohorts disagreed with Step 1 becoming pass/fail. Forty-eight percent of respondents admitted that an applicant’s Step 1 score was previously the most important factor in deciding to interview an applicant. Once the shift to pass/fail occurs, 58.4% of PDs stated that they will focus more on subsequent exams such as Step 2 CK scores as the most important, followed by clerkship grades and then letters of recommendation [9].

A survey study of PDs in diagnostic and interventional radiology as well as nuclear medicine showed that 72.7% of respondents will place more importance on the reputation of the medical school of the applicant in deciding who gets an interview and predicted that access to radiology residency positions will further decrease for osteopathic medical students and international medical graduates [6]. In a study with vascular, thoracic, and integrated plastic surgery PDs, approximately 64% of respondents agreed that medical school reputation will become more important [5].

However, other survey studies in fields such as anesthesiology showed that only 31.1% of PDs believe that Step 2 CK will become more important after Step 1 becomes Pass/Fail. Furthermore, some of the factors that were deemed the least important included clerkship grades and research accomplishments [7].

Dermatology is considered to be the second least diverse field in medicine after orthopedic surgery [3]. According to the US Census, more than half the population of the country is projected to belong to a minority group by 2044. The shift to pass/fail was designed in part to help underrepresented groups match into dermatology to meet the increasing diversification of the United States. However, dermatology, which is consistently one of the most competitive fields to match into, had one of the lowest match rates according to recent National Resident Matching Program (NRMP) data. 2020 NRMP data shows the average Step 1 score of MD seniors matching into dermatology was between 251 and 260, with 92.5% of these students having five or more research abstracts or publications [11,12]. In addition, a study showed that there was a significant correlation between MDs matching into dermatology and research experience. There was no significant correlation between DOs matching and research experiences [4]. This study is an ancillary report from a collective survey regarding PDs across all specialties [5]. This current study was designed to determine the perspectives of dermatology PDs regarding Step 1 pass/fail as they decide which applicant gets an interview.

## Materials and methods

After receiving Institutional Review Board (IRB) exemption status, we selected participants from 144 Accreditation Council for Graduate Medical Education (ACGME) including 27 former American Osteopathic Association (AOA) dermatology programs, using the contact information found from their respective online databases. An eight-item survey was constructed on a three-point Likert scale along with one free text response and four demographic questions asking participants about their gender, age, position, and number of years in that position.

The Likert items, free-text responses, and demographic questions were similar to those of a previous study done with radiology PDs across the country and their attitudes on Step 1 becoming pass/fail [6]. The survey can be found in the Appendix.

The anonymous survey was sent out over the course of three weeks in June 2021 with weekly individualized reminder requests for voluntary participation. Data was collected and analyzed using Statistical Product and Service Solutions (SPSS) (IBM SPSS Statistics for Windows, Version 24.0, Armonk, NY).

## Results

Of the 144 surveys sent, 35 participants (24%) responded; 28 (80%) were residency PDs, three (8.6%) were assistant residency PDs, three (8.6%) were the program coordinators, and one (2.8%) was an associate professor.

The average time serving in their respective positions was nine years. Regarding the age of participants, two (5.8%) were less than 30 years old, six (17.6%) were aged 30-39, 11 (32.3%) were aged 40-49, seven (20.5%) were aged 50-59, and eight (23.5%) were 60 or older. One responder did not reveal their age. Sixteen (45.7%) participants identified as female, 17 (48.5%) identified as male, and two preferred not to answer. Of the 35 respondents, 22 completed the survey in full and 13 were incomplete.

Among the respondents, 40.9% either agreed or strongly agreed that Step 1 was previously the most important metric when determining whether an applicant would receive an interview invitation. Further, 63.6% either disagreed or strongly disagreed that Changing Step 1 to pass/fail was objective, and 63.63% of responders either agreed or strongly agreed that their program would respond to the change by requiring Step 2 CK after Step 1 Pass/Fail before offering a residency interview. In addition, 59.1% of responders either disagreed or strongly disagreed that the change will make it more difficult for students to match into their respective dermatology program, and 54.5% either disagreed or strongly disagreed that the change would make it more difficult for osteopathic medical students to match into their respective dermatology program. The results also showed that 63.6% of responders either disagreed or strongly disagreed that the change will help alleviate racial and socioeconomic disparities during the residency application process. The change will make it more challenging to predict academic success during residency, according to 72.7% of respondents who either agreed or strongly agreed with this statement. Finally, 54.5% of respondents either disagreed or strongly disagreed that Changing Step 1 to pass/fail was reasonable. Refer to Table 3 in the Appendices for the full results of the Likert Scale questionnaire.

Table 1 shows the three top-ranked and lowest-ranked factors of an application that received the highest consensus among completed surveys before the change to pass/fail. Refer to Table 4 in the Appendices for full results.

**Table 1:** Before Step 1 became pass/fail USMLE: United States Medical Licensing Examination COMLEX: Comprehensive Osteopathic Medical Licensing Examination

Rank	Ranked Factors (N=23)
Highly ranked aspects of the application	60.8% of responders had “Letters of Recommendation” in their top 3. Ranked as the most important 17.3% of the responses
	42.8% of responders had “Completing Audition Rotation at the Program” in their top 3. Ranked as the most important 33.3% of the responses
	40.9% of responders had “USMLE Step 1 Scores” in their top 3. Ranked as the most important 13.6% of the responses
Lowly ranked aspects of the application	54.5% of responders had “COMLEX Level 1 and 2 Scores” in their lowest 3. Ranked as the least important in 36.3% of the responses
	45.4% of responders had “Contribution to Diversity Goals” in their lowest 3. Ranked as the least important in 13.6% of the responses
	33.3% of responders had “Alpha Omega Alpha (AOA) Honor Society Status” in their lowest 3. Ranked as the least important in 19.1% of the responses

Table 2 shows the three top and lowest-ranked factors of an application that received the highest consensus among completed surveys after the pass/fail change. Refer to Table 5 in the Appendices for full results.

**Table 2:** After Step 1 becomes pass/fail USMLE: United States Medical Licensing Examination

Rank	Ranked Factors (N=23)
Highly ranked aspects of the application	54.5% of responders had “Letters of Recommendation” in their top 3. Ranked as the most important in 18.2% of the responses
	45% of responders had “Completed Audition Rotation at Program” in their top 3. Ranked as the most important in 35% of the responses
	38.1% of responders had “USMLE Step 2 CK Scores” in their top 3. Ranked as the most important in 9.5% of the responses
Lowly ranked aspects of the application	45% of responders had “Alpha Omega Alpha (AOA) Honor Society Status” in their lowest 3. Ranked as the least important in 15% of the responses
	42.8% of responders had “Medical School Reputation” in their lowest 3. Ranked as the least important in 14.2% of the responses
	38.1% of responders had “Contribution to Diversity Goals” in their lowest 3. Ranked as the least important in 14.3% of the responses

## Discussion

The majority (63.6%) of dermatology PDs and other respondents disagreed that changing Step 1 to pass/fail is reasonable or objective. Concurrently, the Step 1 examination outcome was not the most important factor in determining interview eligibility before Step 1 became pass/fail. Among the respondents, 63.6% stated that their program would now require Step 2 CK before considering extending an interview to an applicant. A possible conjecture is that Step 2 CK examination scores now carry the same, if not more weight as Step 1 scores in terms of distinguishing between applicants. However, the data suggest that 40.9% of respondents had Step 1 in their top three pre-pass/fail, with 38.1% of responses having Step 2 CK being in their top three post-pass/fail policy. In addition, 13.6% of respondents stated that Step 1 was the most important pre-pass/fail factor, which decreased to 9.5% of respondents stating that Step 2 would be the most important post-pass/fail. The top three percentages for both clerkship grades and research decreased slightly as well.

Completing an audition rotation at the program was in the top three for both pre- and post-pass/fail surveys. This factor increased in importance, from 42.8% to 45% of respondents placing it in their top three. The frequency of personal statements being in the top three increased from 30.4% to 36.3%. The decreasing percentages seen for Step 2 CK, clerkship grades, and research in contrast to the increase in percentages seen for audition rotations and personal statements suggest that directors are open to adopting a more holistic approach to screening applicants.

Notably, 72.7% of responders believed that changing Step 1 to pass/fail will increase the difficulty of predicting who will be successful in residency. If programs are now relying more on audition rotations, this could suggest that although rotating at a program will increase an applicant’s chance of getting accepted, obtaining that rotation in the first place will become much more difficult because rotation spots are limited.

Invitational Conference on USMLE Scoring has stated that the rationale for pass/fail is manifold, with one reason being to address racial and socioeconomic disparities. Although it is a worthwhile goal, 63.6% of responders do not think the examination policy change will provide social relief. Furthermore, “Contribution to Diversity Goals” was the only item on both ranking surveys that never appeared in the top three. Free text responses provided by some respondents further supported this sentiment. Refer to Table 6 in Appendices for all free text responses.

In terms of the osteopathic match, roughly half of the respondents agreed that DO students will have a more difficult time matching into dermatology. Approximately 50% of respondents agreed that all medical students will have more difficulty matching dermatology. The relatively even split of these two survey items suggests that although certain aspects of an application can have increasing importance, how that relates to the relative ease of obtaining a dermatology residency remains unclear.

## Conclusions

There has been much speculation and many questions and concerns regarding how the transition of Step 1 to pass/fail will affect a student’s chances of successfully matching into a competitive specialty. Before a definitive match outcomes list is examined, definitive results are difficult to generate. A literature review has shown that fields such as otolaryngology will highly focus on Step 2 CK scores, anesthesiology will focus more on clerkship grades, and radiology and surgical fields will focus more on medical school reputation. Based on the survey study, dermatology PDs seem to want to focus more on letters of recommendation, audition rotations, and Step 2 CK. Because each field seems to prioritize different aspects of an application, students should attempt to gain as much exposure to different fields through research and shadowing to narrow down their ideal specialties during their first and second years of medical education. Knowing early on what parts of an application certain specialty desires will help the student have more time to carefully tailor their applications in the third year.

## Appendices

**Table 3:** Likert scale survey questions USMLE: United States Medical Licensing Examination CK: Clinical Knowledge

Statement	STRONGLY DISAGREE	DISAGREE	AGREE	STRONGLY AGREE	TOTAL
USMLE Step 1 scores were previously the most important metric when determining whether an applicant received an interview invitation.	27.27%	31.82%	22.73%	18.18%	22
Changing Step I to Pass/Fail is more objective.	18.18%	45.45%	22.73%	13.64%	22
Changing Step 1 to Pass/Fail, our program will now require Step 2 CK before an interview is offered to a residency candidate.	4.55%	31.82%	36.36%	27.27%	22
Changing Step 1 to Pass/Fail will make it more difficult for students to match into our dermatology program.	9.09%	50.00%	18.18%	22.73%	22
Changing Step 1 to Pass/Fail will make it more difficult for osteopathic medical students to match into our dermatology program.	18.18%	36.36%	22.73%	22.73%	22
Changing Step 1 Pass/Fail will help alleviate racial and socioeconomic disparities during the residency application process.	18.18%	45.45%	27.27%	9.09%	22
Changing Step 1 to Pass/Fail will make it more challenging to predict academic success during residency.	4.55%	22.73%	45.45%	27.27%	22
Changing Step 1 to Pass/Fail is reasonable.	27.27%	27.27%	36.36%	9.09%	22

**Table 4:** Ranking activity before Step 1 pass/fail USMLE: United States Medical Licensing Examination COMLEX: Comprehensive Osteopathic Medical Licensing Examination CK: Clinical Knowledge AOA: Alpha Omega Alpha Honor Society

	1 (Most Important)	2	3	4	5	6	7	8	9	10	11	12	13 (Least Important)
Medical School Reputation	13.64%	9.09%	0.00%	9.09%	13.64%	4.55%	0.00%	13.64%	0.00%	4.55%	9.09%	13.64%	9.09%
USMLE Step 1 Scores	13.64%	13.64%	13.64%	9.09%	0.00%	13.64%	9.09%	18.18%	9.09%	0.00%	0.00%	0.00%	0.00%
USMLE Step 2 CK Scores	0.00%	0.00%	4.55%	9.09%	9.09%	4.55%	13.64%	13.64%	13.64%	9.09%	9.09%	9.09%	4.55%
COMLEX Scores (Levels 1 and 2)	4.55%	9.09%	0.00%	4.55%	9.09%	0.00%	0.00%	0.00%	13.64%	4.55%	0.00%	18.18%	36.36%
Class Rank	0.00%	0.00%	19.05%	9.52%	9.52%	14.29%	9.52%	4.76%	0.00%	4.76%	19.05%	4.76%	4.76%
Pre-Clerkship Grades (years 1 and 2)	0.00%	4.76%	0.00%	9.52%	4.76%	19.05%	0.00%	0.00%	19.05%	9.52%	19.05%	9.52%	4.76%
Clerkship Grades	9.09%	9.09%	9.09%	13.64%	9.09%	9.09%	13.64%	9.09%	4.55%	0.00%	9.09%	4.55%	0.00%
Research	4.55%	22.73%	4.55%	13.64%	4.55%	18.18%	13.64%	4.55%	9.09%	4.55%	0.00%	0.00%	0.00%
Letters of Recommendation	17.39%	17.39%	26.09%	4.35%	8.70%	4.35%	4.35%	0.00%	4.35%	4.35%	4.35%	0.00%	4.35%
Personal Statement	8.70%	13.04%	8.70%	17.39%	4.35%	4.35%	13.04%	8.70%	0.00%	13.04%	8.70%	0.00%	0.00%
AOA Honor Society Status	0.00%	4.76%	9.52%	0.00%	4.76%	4.76%	0.00%	0.00%	14.29%	28.57%	4.76%	9.52%	19.05%
Completed Audition Rotation at the Program	33.33%	0.00%	9.52%	4.76%	9.52%	0.00%	9.52%	19.05%	4.76%	0.00%	4.76%	4.76%	0.00%
Contribution to Diversity Goals	0.00%	0.00%	0.00%	0.00%	18.18%	4.55%	9.09%	4.55%	4.55%	13.64%	9.09%	22.73%	13.64%

**Table 5:** Ranking activity after Step 1 pass/fail USMLE: United States Medical Licensing Examination COMLEX: Comprehensive Osteopathic Medical Licensing Examination CK: Clinical Knowledge AOA: Alpha Omega Alpha Honor Society

	1 (Most Important)	2	3	4	5	6	7	8	9	10	11	12 (Least Important)
Medical School Reputation	14.29%	9.52%	0.00%	9.52%	0.00%	4.76%	4.76%	4.76%	9.52%	9.52%	19.05%	14.29%
USMLE Step 2 CK Scores	9.52%	19.05%	9.52%	9.52%	14.29%	14.29%	4.76%	9.52%	0.00%	4.76%	4.76%	0.00%
COMLEX Scores (Levels 1 and 2)	4.76%	9.52%	9.52%	4.76%	9.52%	0.00%	4.76%	4.76%	4.76%	0.00%	4.76%	42.86%
Class Rank	0.00%	10.00%	10.00%	25.00%	5.00%	5.00%	5.00%	5.00%	5.00%	25.00%	5.00%	0.00%
Pre-Clerkship Grades (years 1 and 2)	0.00%	5.00%	5.00%	15.00%	10.00%	10.00%	5.00%	5.00%	15.00%	10.00%	20.00%	0.00%
Clerkship Grades	4.76%	9.52%	9.52%	9.52%	23.81%	14.29%	9.52%	4.76%	4.76%	0.00%	4.76%	4.76%
Research	13.64%	4.55%	9.09%	9.09%	9.09%	18.18%	18.18%	9.09%	9.09%	0.00%	0.00%	0.00%
Letters of Recommendation	18.18%	27.27%	9.09%	0.00%	9.09%	9.09%	0.00%	13.64%	4.55%	4.55%	4.55%	0.00%
Personal Statement	9.09%	4.55%	22.73%	9.09%	13.64%	9.09%	9.09%	0.00%	18.18%	4.55%	0.00%	0.00%
AOA Honor Society Status	0.00%	0.00%	15.00%	0.00%	5.00%	0.00%	5.00%	20.00%	10.00%	20.00%	10.00%	15.00%
Completed Audition Rotation at the Program	35.00%	5.00%	5.00%	5.00%	0.00%	10.00%	20.00%	0.00%	0.00%	5.00%	10.00%	5.00%
Contribution to Diversity Goals	0.00%	0.00%	0.00%	9.52%	4.76%	4.76%	9.52%	19.05%	14.29%	9.52%	14.29%	14.29%

**Table 6:** Free text responses COMLEX: Comprehensive Osteopathic Medical Licensing Examination USMLE: United States Medical Licensing Examination

#	Responses
1	I think that this change will disproportionately disadvantage exemplary students from less well-known medical schools and may limit students to programs within geographic proximity of their medical school. As a person who attended a less well-known medical school for family and financial reasons but scored in the 99th percentile on Step 1 opened doors for me that would have likely remained closed otherwise. I also acknowledge that Step 1 has caused significant stress for second-year medical students in the past and that it is an incomplete metric with which to evaluate residency candidates. This is a complex issue and I am not sure there is a “correct” answer. I will be advising my students to aggressively pursue research opportunities, to consider away rotations at institutions where they would like to match, and to work to craft unique and engaging personal statements.
2	Changing to Pass Fail hurts those it implies it would like to help. If prejudices come out, they will not go away by not giving a score. The evaluator’s pre-existing expectations will be manifested even more so. It is pointless and ludicrous.
3	It gives an unfair advantage since COMLEX scores can be used against Dos whereby a pass/fail USMLE score can give MDs a distinct advantage because there will no longer be a gold standard in scores to attain.
4	Hurts applicants from schools without a residency program.
5	Great survey
